# Carboplatin, pemetrexed, and pembrolizumab was effective for primary salivary gland‐type lung adenocarcinoma diagnosed due to esophageal stricture: A case report

**DOI:** 10.1111/1759-7714.14100

**Published:** 2021-08-08

**Authors:** Keita Kawakado, Tomoki Tamura, Masamoto Nakanishi, Go Makimoto, Yumiko Sato, Shoichi Kuyama

**Affiliations:** ^1^ Department of Respiratory Medicine National Hospital Organization Iwakuni Clinical Center Iawkuni‐City Japan; ^2^ Department of Internal Medicine, Division of Medical Oncology and Respiratory Medicine Shimane University Faculty of Medicine Izumo Japan; ^3^ Department of Pathology National Hospital Organization Iwakuni Clinical Center Iawkuni‐City Japan

**Keywords:** carboplatin, case report, pembrolizumab, pemetrexed, primary salivary gland‐type tumor of the lung

## Abstract

Primary salivary gland‐type tumors of the lung are rare, accounting for <1% of all lung tumors. There are few reports on chemotherapy for the treatment of primary salivary gland‐type tumors of the lung. The patient in this report was a 71‐year‐old woman who presented with a chief complaint of dysphagia. Upper gastrointestinal endoscopy revealed an esophageal stricture, but biopsy showed no malignancy. Chest computed tomography (CT) showed carcinomatous lymphangiomatosis and a nodule in the right lung. Bronchoscopy showed a rough mucous membrane of the central bronchi, while biopsy showed adenocarcinoma. The patient was diagnosed with bronchogenic adenocarcinoma and received carboplatin, pemetrexed, and pembrolizumab, which alleviated the esophageal stricture and cancerous lymphangiopathy. However, the adenocarcinoma progressed, and she subsequently received several rounds of chemotherapy. One year after diagnosis, the patient died, and pathological autopsy revealed primary salivary gland‐type tumors of the lung.

## Graphical abstract

Lung adenocarcinoma was diagnosed in this patient who subsequently received carboplatin, pemetrexed and pembrolizumab. The carcinomatous lymphangiomatosis improved. Autopsy revealed the tumor was primary salivary gland‐type lung adenocarcinoma.

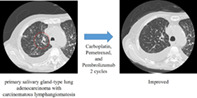

## INTRODUCTION

Primary salivary gland‐type tumors of the lung account for <1% of all lung tumors.^1^ According to the World Health Organization classification of lung tumors, this type includes four major types: adenoid cystic carcinoma, mucoepidermoid carcinoma, myoepithelioma carcinoma, and pleomorphic adenoma.^2^ Surgery, bronchoscopic intervention, and radiotherapy are treatment options for primary salivary gland‐type tumors of the lung.^3^, ^4^ However, there have been few reports on the treatment of primary salivary gland‐type tumors of the lung using chemotherapy.

## CASE REPORT

**FIGURE 1:**
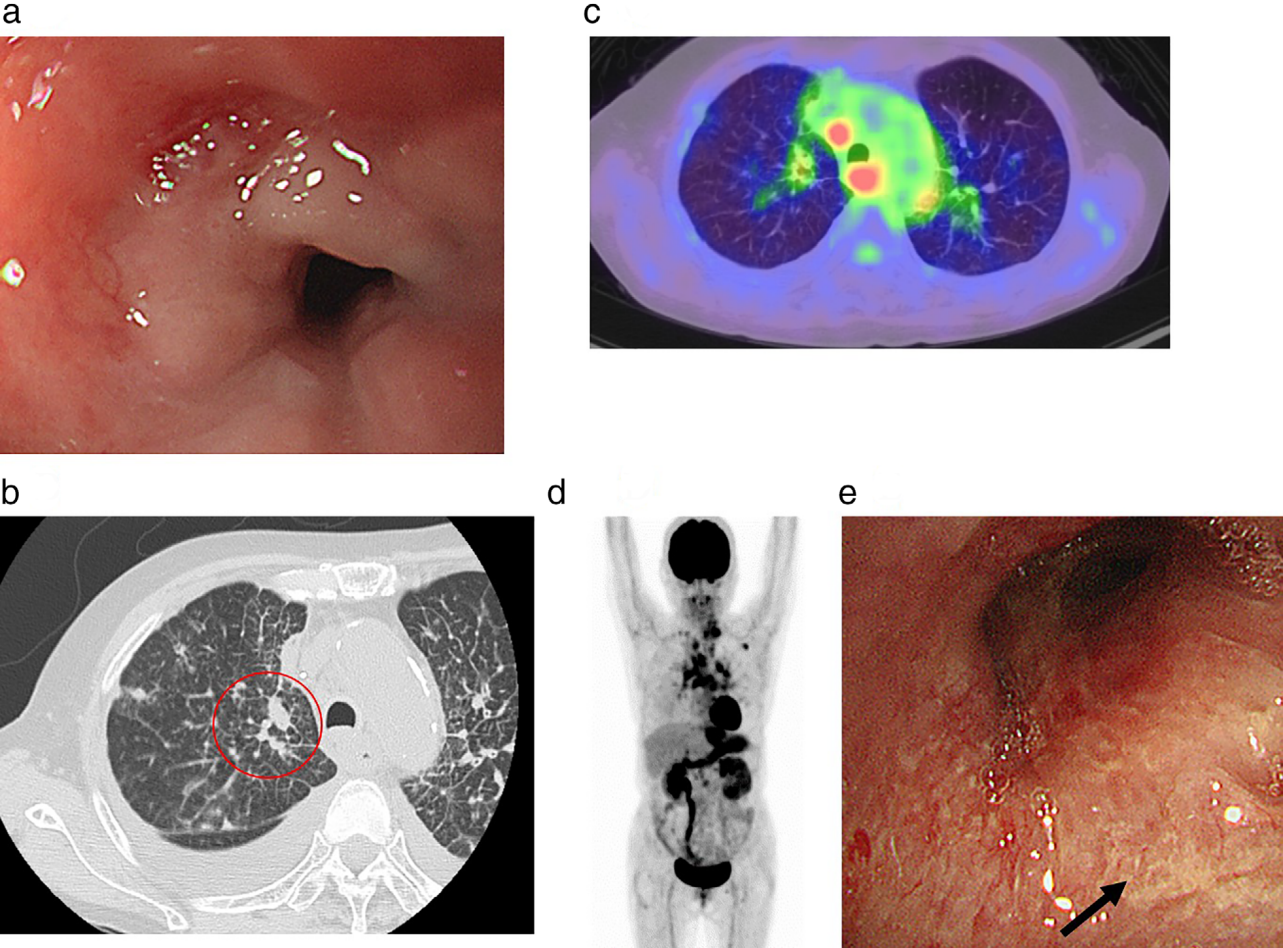
(a) Upper gastrointestinal endoscopy revealed an esophageal stricture. (b) Computed tomography (CT) showed bilateral cancerous lymphangiopathy and a 5 mm nodule in the right lung. (c,d) Positron emission tomography (PET)‐CT showed high accumulation in the lung nodule, thyroid, and cervical, mediastinal, and intraperitoneal lymph nodes. (e) Bronchoscopy showed the mucous membrane of the right upper lobe branch had white mucosal irregularities

**FIGURE 2:**
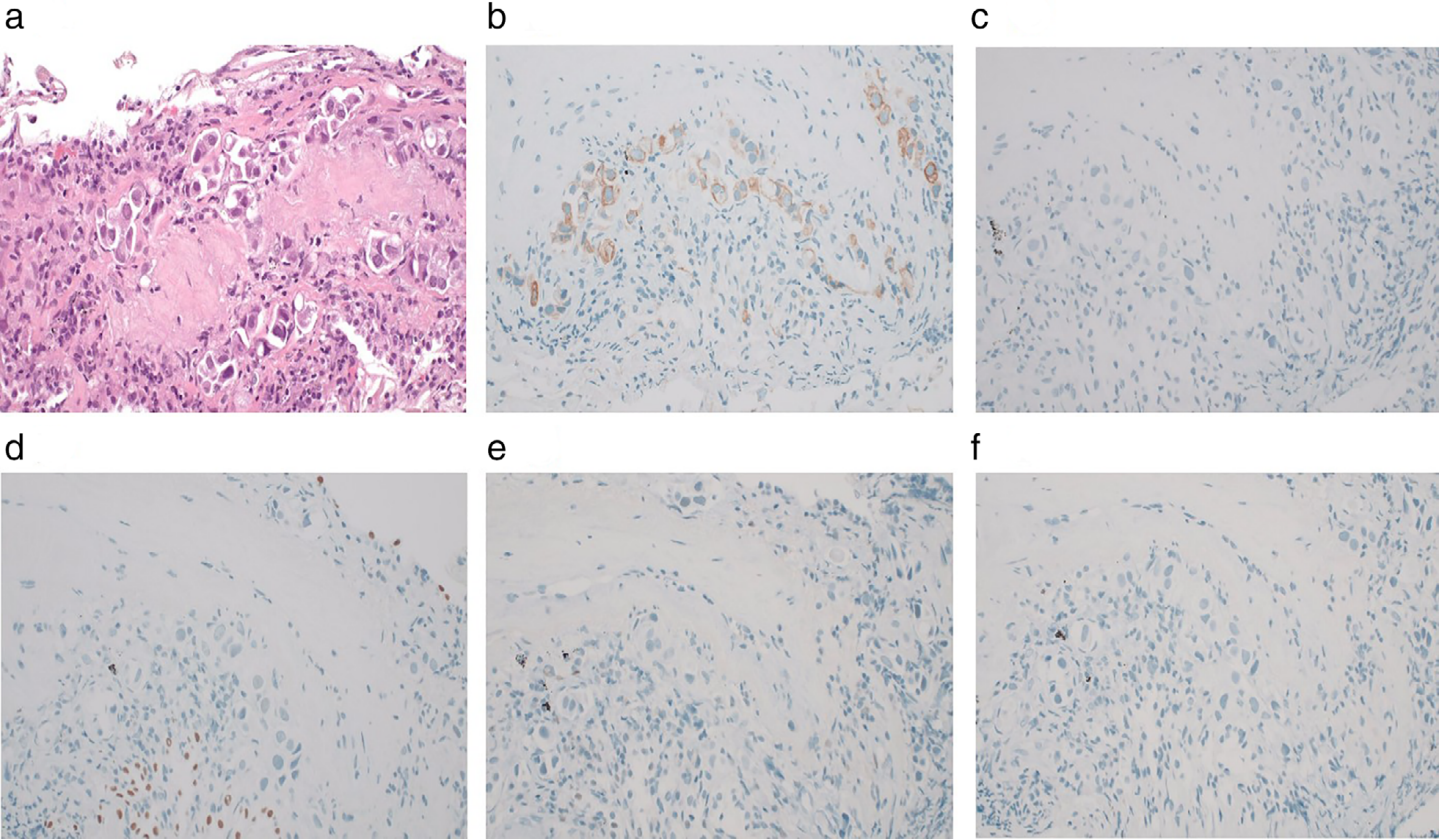
Immunohistochemistry of lung biopsy. (a) Hematoxylin and eosin stain showed adenocarcinoma, (b) CK7 was positive, (c) CK20 was negative, (d) TTF‐1 was negative, (e) ER was negative, and (f) CDX‐2 was negative

**FIGURE 3:**
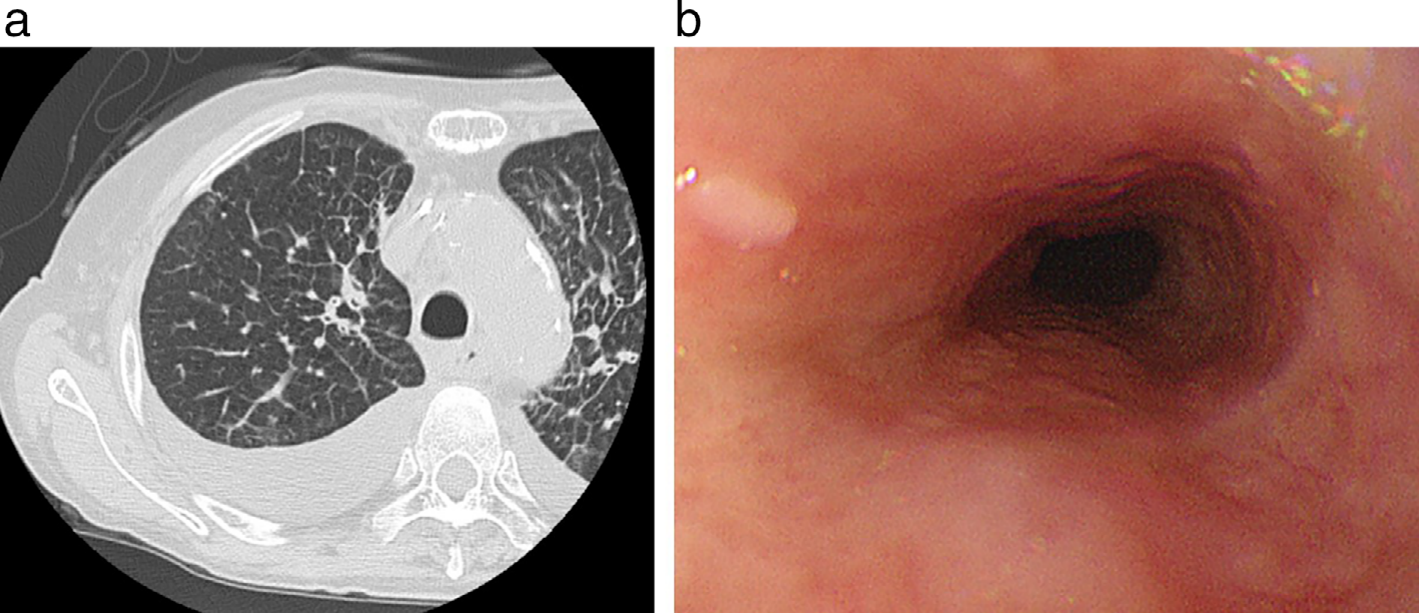
(a) Computed tomography (CT) showed the cancerous lymphangiopathy had improved, and (b) upper gastrointestinal endoscopy showed the esophageal stricture had improved

A 72‐year‐old woman presented to our hospital with a chief complaint of dysphagia. She had a history of smoking 20 cigarettes a day from the age of 20 to 70 years, with unremarkable medical and family history. Upper gastrointestinal endoscopy revealed an esophageal stricture, while biopsy showed no malignancy (Figure 1(a)). Computed tomography (CT) showed bilateral cancerous lymphangiopathy and a 5‐mm right lung nodule (Figure 1(b)). Positron emission tomography (PET)‐CT showed high accumulation in the lung nodule, thyroid, and cervical, mediastinal, and intraperitoneal lymph nodes (Figure 1(c),(d)). On bronchoscopy, the right upper lobe branch mucous membranes had white mucosal irregularities. Biopsy revealed adenocarcinoma (Figure 1(e)). Thyroid biopsy performed to identify the primary lesion, showed metastatic adenocarcinoma. On immunohistochemistry, the lung biopsy sample was positive for CK7, and negative for CK20, TTF‐1, ER, and CDX‐2 (Figure 2). Therefore, the primary lesion was unidentifiable. Bronchoscopic findings led to the diagnosis of bronchogenic adenocarcinoma (cT1aN3M1c cStage IVB). Additional genetic testing revealed that the patient was negative for epidermal growth factor receptor (EGFR), anaplastic lymphoma kinase fluorescence in situ hybridization, c‐ros oncogene 1, v‐Raf murine sarcoma viral oncogene homolog B1, and PD‐L1 22C3 immunohistochemical staining, with a tumor proportion score (TPS) of less than 1%. She received CBDCA, PEM, and pembrolizumab for cancer treatment. After two cycles of CBDCA + PEM + pembrolizumab, CT revealed improved cancerous lymphangiopathy (Figure 3(a)), while upper gastrointestinal endoscopy showed improved esophageal stricture (Figure 3(b)), and evaluation by RECIST revealed stable disease. Before chemotherapy, she was unable to eat using her mouth, but after two cycles, she was able to do so. After four cycles of her treatment, she received two cycles of PEM and pembrolizumab as maintenance. Although the drug efficacy was maintained for five months, the carcinomatous lymphangiomatosis worsened. She received two cycles of tegafur/gimerac/oteracil as second‐line therapy, four cycles of docetaxel and ramucirumab as third‐line therapy, and one cycle of amrubicin as fourth‐line therapy. However, the carcinomatous lymphangiomatosis continued to worsen, and brain metastasis occurred. She died one year after her diagnosis.

**FIGURE 4:**
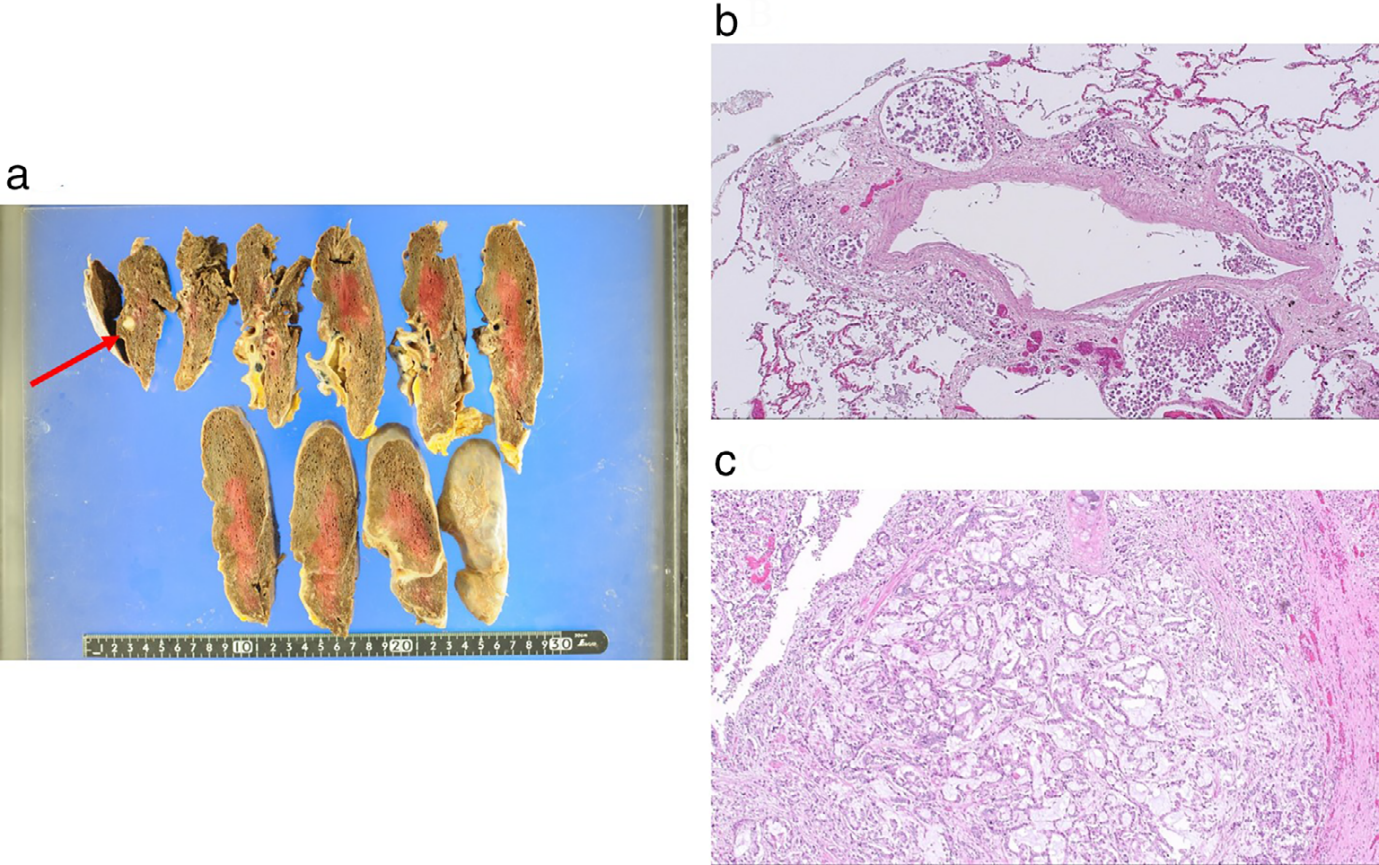
(a) A macro image showed a 5‐mm nodule in the right upper lobe of the lung. (b) There were poorly differentiated tumor cells in the lymph vessels. (c) The lung mucus‐producing atypical epithelium was glandularized and proliferative. A continuous structure resembling the bronchial glands was seen

Pathological examination was performed to reveal the primary tumor pathology. There were 5‐mm nodules in the right upper lobe of the lung (Figure 4(a)). The lymph vessels exhibited poorly differentiated tumor cells (Figure 4(b)). Histologically, the lung mucus‐producing atypical epithelium was glandularized and proliferative. A continuous structure resembling the bronchial glands was observed. Well‐differentiated tumor cells were found in the right upper lobe branch (Figure 4(c)). Organ metastasis in the thyroid, liver, left ventricle of the heart, esophagus, stomach, duodenum, bilateral adrenal glands, peritoneum, and pleura, and lymphatic metastasis, were suspected. Only the right upper lobe of the lung exhibited well‐differentiated tumor cells. The patient was diagnosed with primary salivary gland‐type lung adenocarcinoma.

## DISCUSSION

Primary salivary gland‐type tumors are rare.^1^ They arise from the bronchial submucosal glands and present as exophytic endobronchial tumors in the trachea or mainstem bronchi.^5^ Primary salivary gland‐type tumors have low malignant potential. They occur in the submucosal glands of the central airways.^6^ Surgery, bronchoscopic intervention, and radiotherapy have been reported as treatment options, but there are few reports on treatment with chemotherapy.^3^, ^4^ Although the roles of adjuvant chemotherapy and radiation are not well‐defined, they may be effective in selected cases, especially tumors with aggressive biological behavior.^7^ The overall survival at 3, 5, and 10 years has been reported to be 91.3%, 86%, and 80.6%, respectively. Disease‐free survival was 90.1%, 78.6%, and 55%, respectively. The disease progresses slowly and is typically diagnosed during the earlier stages. Among patients diagnosed with primary salivary gland‐type tumors of the lung, 69.1% were diagnosed at stage I, 18.2% were diagnosed at stage II, 10.2% were diagnosed at stage III, and 2.3% were diagnosed at stage IV. The main treatment option is surgery (95.5%), and chemotherapy was administered in only 4.5% of patients.^6^ Icotinib, an EGFR tyrosine kinase inhibitor, has been previously used for *EGFR* mutation‐positive primary salivary gland‐type tumors of the lung. It was found to be temporarily effective, but became less effective after three months.^8^

In the case reported here, the primary lesion was not identified, and bronchoscopic findings led to a diagnosis of bronchogenic adenocarcinoma. Based on the treatment guidelines of lung cancer, CBDCA, PEM, and pembrolizumab were administered. The treatment alleviated dysphagia, which was probably caused by a reductant of the esophageal tumor, and chest CT showed carcinomatous lymphangiomatosis improvement. The primary lesion in this case was probably in the right upper bronchi because well‐differentiated tumor cells were observed in the right upper bronchi. The other organs exhibited poorly differentiated tumor cells. Poorly differentiated adenocarcinomas can be diagnosed by prenatal pathological and autopsy findings. A well‐differentiated mucus‐producing adenocarcinoma similar to the bronchial gland was found in the nodule of the upper right lobe, with spread to surrounding poorly differentiated components. Additionally, as we could not identify another primary lesion, we considered this could be a progression from the right upper bronchi. The patient was a smoker, the greatest and most preventable risk factor for cancer. Carcinogens present in particulate and gas‐phase smoke can promote tumor growth, and metastasis by influencing the cell cycle.^9^

To our knowledge, this is the first report on combination therapy with immune checkpoint inhibitors and chemotherapy. No long‐term effects were observed, and it improved the patient's quality of life.

Here, we report the effective treatment of primary salivary gland‐type lung adenocarcinoma with CBDCA, PEM, and pembrolizumab. Combination therapy with immune checkpoint inhibitors and chemotherapy should therefore be a consideration. However, more case reports are needed to further understand this rare disease.

## CONFLICT OF INTERESTS

This research did not receive any specific grant from funding agencies in the public, commercial, or not‐for‐profit sectors.
